# The effect of cognitive effort on the sense of agency

**DOI:** 10.1371/journal.pone.0236809

**Published:** 2020-08-12

**Authors:** Eva Van den Bussche, Maryna Alves, Yannick P. J. Murray, Gethin Hughes

**Affiliations:** 1 Brain & Cognition, KU Leuven, Leuven, Belgium; 2 Health Psychology research group, KU Leuven, Leuven, Belgium; 3 Department of Psychology, University of Essex, Colchester, United Kingdom; McGill University, CANADA

## Abstract

While we are performing a demanding cognitive task, not only do we have a sense of cognitive effort, we are also subjectively aware that we are initiating, executing and controlling our thoughts and actions (i.e., sense of agency). Previous studies have shown that cognitive effort can be both detrimental and facilitative for the experienced sense of agency. We hypothesized that the reason for these contradictory findings might lie in the use of differential time windows in which cognitive effort operates. The current study therefore examined the effect of cognitive effort exerted on the current trial, on the previous trial or across a block of trials on sense of agency, using implicit (Experiment 1) and explicit (Experiment 2) measures of sense of agency. We showed that the exertion of more cognitive control on current trials led to a higher explicit sense of agency. This surprising result was contrasted to previous studies to establish potential reasons for this surprising finding and to formulate recommendations for future studies.

## Introduction

Intuitively, we all grasp the concept of cognitive effort. We all know what it is like to feel that a cognitive task is demanding and effortful. However, defining cognitive effort is not straightforward [1]. Cognitive effort refers to the degree of engagement in demanding cognitive tasks, as opposed to tasks that can be completed using routine or habitual behavior, which require little effort. An influential neuroeconomics approach to effort postulates that deciding whether to invest cognitive effort comes down to investigating the relevant costs and benefits (see for example [1]). More specifically, several theories have proposed that effort may be primarily implicated in the decision to engage cognitive control resources ([2–5]; for a review see [1]).

The broad concept of cognitive control comprises cognitive operations such as planning a new strategy, evaluating it, controlling its execution and correcting possible errors. It kicks in when routine activation of behavior is no longer sufficient for optimal performance [6]. Cognitive control thus allows us to perform intelligent, purposive behavior. When performing basically any task or when we aim to achieve a goal, we need to stay focused and inhibit dominant, yet irrelevant information to prevent being distracted from our task. Whenever an irrelevant source of information interferes with our task performance, we will exert additional cognitive control in order to preserve task performance and resolve conflict. Conflict can be defined as the simultaneous activation of incompatible representations [7]. In an experimental context, conflict tasks such as the Eriksen flanker task are often used to measure cognitive control [8]. This task consists of a central target arrow flanked by distractor arrows. Congruent (i.e., flankers and target point in the same direction) and incongruent (i.e., flankers and target point in opposite directions) stimuli are presented and participants must indicate the direction of the central target arrow as fast and as accurately as possible. Responses to incongruent trials are slower and are more error prone than to congruent trials. This difference in performance between incongruent and congruent trials constitutes the flanker effect. Here, dominant yet task irrelevant stimuli (i.e., flanker arrows) need to be suppressed in order to perform this task well.

When people encounter interference, such as conflict as defined above, cognitive control allows them to adjust their behavior to overcome it. However, exerting cognitive control comes at a cost and this is where cognitive effort comes in. The neuroeconomic theories treat cognitive effort as the opportunity cost of exerting cognitive control (i.e., the decision to expend cognitive control is accompanied by the missed benefit of avoiding effort; see [1] for a review). Cognitive control usually drives cognitive effort and exerting more cognitive control would lead to a higher effort cost.

While we are performing a demanding cognitive task, not only do we have a sense of cognitive effort, we are also subjectively aware that we are initiating, executing and controlling our thoughts and actions. The feeling of being in charge when we perform voluntary actions is called the sense of agency [9]. The brain appears to actively construct the sense of agency using motor actions, sensory feedback, previous experiences, cause-and-effect inferences, and so on [10,11]. The core of sense of agency is the association between a voluntary action (e.g., pressing a light switch) and an outcome (e.g., the light in the room goes on). Experimentally, sense of agency can be explicitly measured by simply asking participants to judge whether their action caused an outcome. Alternatively, implicit measures can be used, such as the compression of perceived time between action and outcome [12]. In the intentional binding paradigm, participants judge the perceived time of a voluntary action or a subsequent outcome. It has been shown that voluntary actions (as compared to involuntary movements) are perceived as shifted in time towards their subsequent outcomes and that the outcomes are perceived as shifted towards the voluntary actions that caused them. Based on this implicit measure, sense of agency is defined as the compression (or underestimation) of the perceived time interval between action and outcome.

Crucially, effort and sense of agency have been linked to each other, dating back even to the 19^th^ century (for an overview see [13]). It has been argued that without the subjective experience of effort there could not be any feeling of agency or causality, but only mere facts of behavior. According to this view, it is the conscious experience of effort that makes self-knowledge possible. If effort is a crucial determinant of sense of agency, then experimental manipulations of cognitive effort should also influence the accompanying sense of agency. A few studies have shown that sense of agency can indeed be influenced by increased cognitive effort.

Howard et al. instructed participants to memorize two (low effort) or eight (high effort) letters [14]. After encoding, a maintenance period started by a self-initiated action which ended after a variable duration with an outcome (i.e., a tone). Participants’ estimation of the duration of this maintenance period was used to measure intentional binding. The results showed that a high cognitive effort context decreased intentional binding compared to a low cognitive effort context. Similar results were obtained by Hon et al. [15] who used an explicit instead of an implicit measure of sense of agency. Contrarily, Demanet et al. [16] observed increased intentional binding in a high physical effort context. In the study of Sidarus and Haggard [17], participants responded to flanker trials (i.e., the action) which triggered the appearance of a colored circle after a variable delay (i.e., the outcome). They judged how much control they felt over the colored circles that were triggered by their actions. Results indicated that incongruent flanker trials led to lower sense of agency compared to congruent and neutral flanker trials. Vastano et al. reached the same conclusion using an implicit intentional binding measure of sense of agency [18]. However, although Wang et al. [19] also found that sense of agency ratings were lower for incongruent trials, this appeared to be the case only when the previous trial was a congruent trial and thus did not contain conflict. Similarly, Di Costa et al. [20] observed that sense of agency measured with intentional binding is increased *after* making an error.

Thus, previous studies have shown that cognitive effort can be both detrimental and facilitative for the experienced sense of agency. We hypothesize that the reason for the nuances in these findings might lie in the use of differential time windows in which cognitive effort operates. Indeed, the findings seem to depend on whether the effort that was taken into account was exerted on the current trial, the previous trial or across a block of trials. When the *current* trial contained a high level of effort (e.g., incongruent or error trial), sense of agency on that trial decreased [17–19]. However, sense of agency seemed to be increased on trials *following* high effort [19–20]. In *contexts* with high effort, the results are mixed: some studies found increased sense of agency [16] while others observed decreased sense of agency [14–15]. Sidarus and Haggard [17] argued that when an increase of effort can be anticipated, the required cognitive control and expected self-engagement in the task can become part of action planning and prediction. In contrast, effort investment that is sudden and unexpected, for example triggered by unexpected or unpredictable conflict, cannot be predicted and included in action planning. Based on this, we speculate that when high effort is required unpredictably or unexpectedly (e.g., on the current trial), sense of agency will decrease; contrarily, when high effort is anticipated (e.g., after a conflict or in a high conflict context), it should be accompanied by increased sense of agency. We formulated three specific hypotheses. First, in a block of trials where effort is required unpredictably, encountering a high-effort trial will lead to a decreased sense of agency *on that same trial* compared to a low-effort trial. Second, in a block of trials where effort is required unpredictably, encountering a high-effort trial will lead to an increased sense of agency *on the next trial* compared to a low-effort trial. Third, in a block of trials where effort can be anticipated (i.e., frequent high-effort trials), sense of agency will be increased compared to a block of trials where effort cannot be anticipated (i.e., scarce high-effort trials).

In order to assess these hypotheses we will use a flanker task to manipulate conflict and hence the required cognitive control and, in turn, the required effort (i.e., on congruent trials, no conflict is present and thus the required cognitive control and the effort cost is minimal; contrarily, on incongruent trials, conflict is present and thus the required cognitive control and effort cost is larger). In Experiment 1 sense of agency will be measured implicitly using the intentional binding paradigm.

## Experiment 1

### Method

#### Participants

Sixty participants were recruited through the Experiment Management System of the Katholieke Universiteit Leuven. They received course credit for participation. All participants provided written informed consent. All of them had normal or corrected-to-normal eyesight, were not colorblind and were able to operate a keyboard and mouse. We used the following exclusion criteria for participants: response times and/or intentional binding exceeding 2.5 *SD*, and/or error rates above 20%. However, none of the participants met these exclusion criteria. Thus, all 60 participants were included for the analyses (6 males, mean age = 18.52, *SD* = 1.13, range 18–26). This study was approved by the Social and Societal Ethics Committee (SMEC) from KU Leuven (G-2019 01 1493). The study was also preregistered on the Open Science Framework (OSF, osf.io; doi:10.17605/OSF.IO/EM3GQ) and the raw data can be retrieved from https://osf.io/em3gq/ (doi:10.17605/OSF.IO/EM3GQ).

#### Apparatus

The experiment was administered in a computer room in small groups. Stimuli were presented on a 22” monitor with a refresh rate of 60 Hz located approximately 60 cm from the participant. Stimulus presentation and response registration were controlled by PsychoPy v.3.1.0 (Psychology software for Python; [21]).

#### Design and procedure

**Fig 1:**
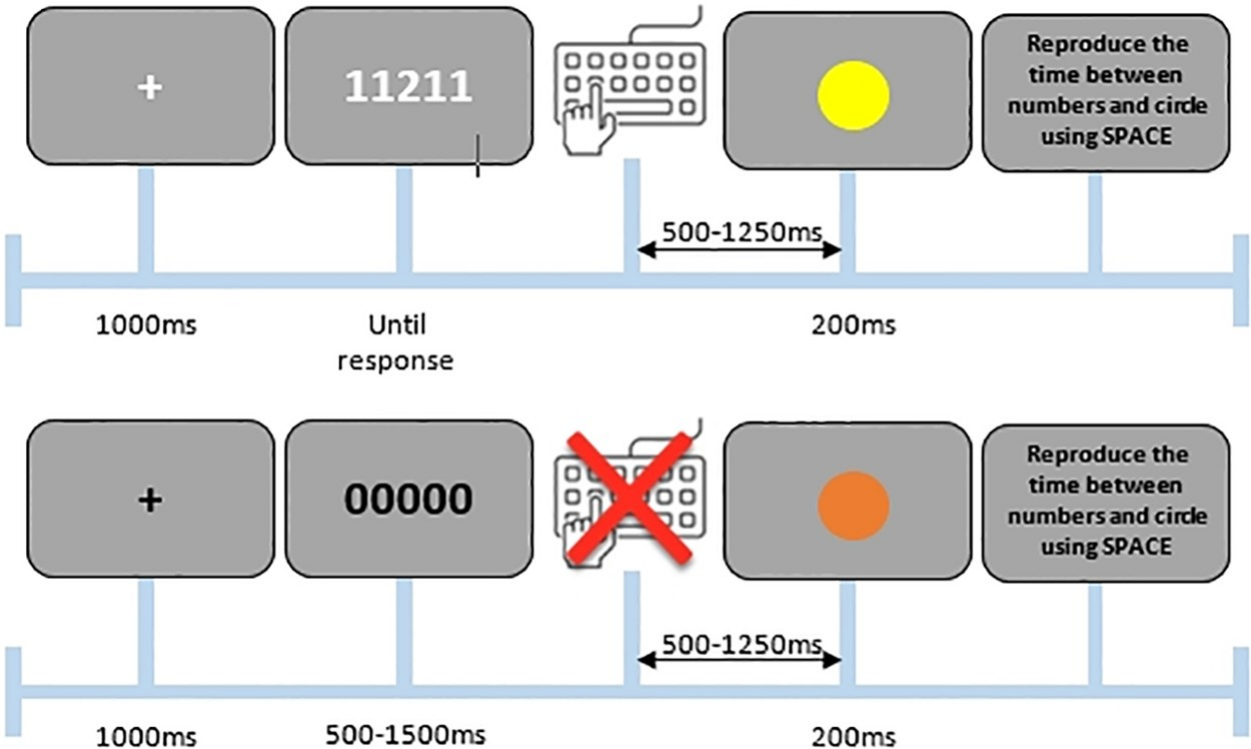
Example of an active (top) and passive (bottom) trial in Experiment 1.

The experiment was composed of active and passive trials. During *active trials* an Eriksen flanker task [8] was used in which a string of numbers was presented in white against a grey background in the center of the screen on each trial (font = Consolas, height = 1.2 deg). A central target number was flanked by two distractor numbers on both sides. Participants were instructed to respond as fast and as accurately as possible to the central target arrow and to ignore the flankers. The flanker stimuli could either trigger the same response as the target (i.e., congruent trials; e.g., “22222”), or trigger a different response as the target (i.e., incongruent trials; e.g., “33233”). The stimuli used were 1, 2, 3 and 4 [22]. Participants had to respond by pressing the corresponding key (1, 2, 3 or 4) on an Azerty keyboard. Specifically, the keys of interest were indicated by stickers: sticker of the number “1” was placed on the “d” key, a “2” on the “f” key, a “3” on the “j” key and a “4” on the “k” key. To speed up response time, participants were instructed to keep their left middle finger on the “1”, their left index finger on the “2”, their right index finger on the “3” and right middle index finger on the “4”. Active trials were announced by a white fixation cross (1000ms) that was followed by the flanker stimulus until a response was provided. After a variable delay of between 500 and 1250ms (in steps of 250ms; [14]), a colored circle was presented on the screen for 200ms (size = (2, 2) deg). The color of the circle depended on the response made on the current trial. Specifically, different colors were linked to passive trials (see below) and to button presses on “1”, “2”, “3” and “4”, leading to five colors (i.e., RGB color space values of pink [1.000, -1.000, 1.000], yellow [1.000, 1.000, -1.000], cyan [-1.000, 1.000, 1.000], orange [1.000, 0.000, -1.000] and blue [-1.000, -1.000, 1.000]). Thus, on active trials an action (i.e., a button press) triggered the appearance of a colored circle, whereas on passive trials no action triggered the appearance of a colored circle. Note that we avoided the use of green and red to prevent any associations with correct/incorrect connotations. Which color was related to which response was randomized across participants. After the disappearance of the circle, participants performed an Interval Reproduction Task [23] in which they reported the estimated length of the delay between the disappearance of the flanker stimulus and the appearance of the circle. They responded by continuously pressing the space bar for the estimated duration. For a schematic overview of an active trial, see Fig 1.

During *passive trials* a string of neutral stimuli (i.e., “00000”) was presented in black against a grey background in the center of the screen. Participants were instructed that these trials did not require a response. As participants did not respond to these stimuli, they were therefore non-agentic [14]. If participants did respond to a passive trial, this was registered as an error. Passive trials were announced by a black fixation cross (1000ms) followed by a neutral stimulus (i.e., “00000”) that was presented for a duration jittered between 500 and 1500ms. After a variable delay of between 500 and 1250ms a colored circle was again presented. Next, participants again had to perform the Interval Reproduction Task in which they estimated and reproduced the length of the delay between the neutral stimulus disappearance and the circle appearance. For a schematic overview of a passive trial, see Fig 1. Note that on passive trials both the fixation cross and the neutral stimulus were presented in a different color (i.e., black) compared to the active trials (i.e., white) to make the non-agentic nature of these trials very clear to the participants.

The experiment started with several practice phases. First, participants received the instructions about the active trials and practiced only the flanker task for 10 trials (five congruent and five incongruent trials). Next, instructions were provided about the presence of the passive trials and participants practiced this on 10 trials where both active and passive trials were presented (six passive, two congruent and two incongruent trials). During these two practice phases, participants received feedback about their responses (i.e., “correct” or “wrong” and their response time in seconds). After this, participants were informed that a circle would appear after the disappearance of the flanker or neutral stimulus and that the color of this circle would depend on their response. They received instructions about the Interval Reproduction Task. Finally, they were also told that after each block they would receive a few additional questions. The first question assessed the level of experienced effort after each block (i.e., “how much effort did you have to exert during the previous block?”). Three additional questions assessed the subjective feeling of agency over congruent, incongruent and passive trials, respectively, during the previous block. For each type of trial (e.g., congruent trials), the trials were listed on the screen (e.g., 11111, 22222, 33333 and 44444) and participants were asked: “To what extent did you have the feeling that you caused the colored circle to appear on the following trials?”. For all of these post-block questions, participants indicated their response using a continuous rating scale ranging from “very little” to “a lot” by a mouse button click on the selected location on the rating scale. In a final practice block, they practiced the full procedure for 10 trials (4 passive, 3 congruent and 3 incongruent trials).

After these practice phases, the experimental phase began where feedback was no longer provided. Active and passive trials were presented intermixed and the inter-trial interval was 1000ms. Experimental trials were divided into three blocks, each containing 216 trials. The blocks varied depending on the ratio of incongruent, congruent, and passive trials: one block with ratio 4:1:1 (i.e., MI block), one with ratio 1:4:1 (i.e., MC block), and one 5:5:2 (i.e., EQ block) ratio of incongruent to congruent to passive trials. The block that had mostly incongruent trials (MI block) served as the high effort context and the bock that had mostly congruent trials (MC block) served as the low effort context [24]. The block with an equal ratio of congruent and incongruent trials (EQ block) served as the block where effort was required unpredictably, to assess the impact of current and previous trial effort on sense of agency. The order of trials in each block was randomized. The order of the blocks was counterbalanced across participants. Finally, after each block, participants received the additional questions to assess exerted effort and experienced agency over each trial type, as described above. In between blocks, participants could take a self-paced break. The whole experiment did not take more than one hour. The experiment was followed by a short debriefing explaining the goal of the study.

#### Statistical analysis

Active trials exceeding 2.5 *SD* of the overall mean RT were excluded from all analyses (1.7% of trials). For RT and intentional binding analyses, erroneous flanker trials were removed (5.9% of trials). For trial-by-trial analyses assessing hypotheses 1 and 2, the first trial (0.46% of trials) and trials following an erroneous flanker trial (5.8% of trials) were also excluded in the EQ block. No trials met our exclusion criterion with regards to intentional binding (i.e., trials exceeding 2.5 *SD* of the overall mean intentional binding).

First, we calculated the measure of intentional binding for active and passive trials based on the Interval Reproduction Task. Specifically, for active trials, the participant’s estimated delay (in milliseconds) was subtracted from the actual delay between their response on the flanker stimulus (i.e., response) and the appearance of the colored circle (i.e., outcome) for each trial (i.e., actual delay—estimated delay). For the passive trials, we subtracted the participant’s estimated delay from the actual delay between the disappearance of the neutral stimulus and the appearance of the colored circle for each trial. Note that a value of zero indicates a perfect estimate, and hence no intentional binding. Positive values indicate an underestimation of the time period, and thus intentional binding. As a control check, we assessed whether our manipulation of intentional binding was successful by looking at the difference in average attentional binding between active (i.e., congruent and incongruent) and passive trials using a paired-samples *t*-test. If our task was indeed successful in eliciting intentional binding, we should observe that intentional binding in active trials was larger than in passive trials (where no action is present). Note that comparing active (i.e., action) and passive (i.e., no-action) trials is an established paradigm in this field (e.g., [23,25,26]).

Second, to test hypotheses 1 and 2, we focused on the EQ block where effort is required unpredictably (i.e., equal proportion of incongruent and congruent trials). For hypothesis 1, we examined whether encountering a high-effort trial leads to a decreased sense of agency *on that same trial* compared to a low-effort trial. For hypothesis 2, we examined whether encountering a high-effort trial leads to an increased sense of agency *on the next trial* compared to a low-effort trial. We conducted a 2x2 repeated measures ANOVA analysis with Current and Previous trial congruency as within-subjects factors (both with 2 levels: congruent and incongruent) on intentional binding (in ms), RTs (in ms) and error rates (in %) as (separate) dependent variables.

Third, to test hypothesis 3, we examined whether sense of agency was increased in a block of trials where effort can be anticipated (i.e., MI block), compared to a block of trials where effort cannot be anticipated (i.e., MC block). For this purpose, a block analyses was conducted. We performed a 2x2 repeated measures analysis with Block (2 levels: MC and MI) and Current trial congruency (2 levels: congruent and incongruent) as within-subjects factors on intentional binding, RTs and error rates as (separate) dependent variables.

Note that in the analyses described above, we did not pool all trials across blocks to assess the general effect of current trial congruency on intentional binding. As the different contexts that were created by manipulating the proportion of congruent trials (i.e., EQ, MC and MI) trigger different cognitive control mechanisms, and hence differential congruency effects (see for example [22]), we a priori decided not to pool across blocks. However, an additional exploratory repeated measures analysis across all blocks with current trial congruency as within-subjects factor revealed that the main effect of congruency on intentional binding was not significant (*F*(1,59) = 0.58, *p* = .46, *η*^2^_p_ = 0.010).

Finally, we looked at the subjective ratings of experienced sense of agency and cognitive effort reported after each block for exploratory purposes. We conducted a repeated measures analysis with block (3 levels: EQ, MC or MI) as within-subjects factor on the reported experienced effort after each block and a repeated measures analysis with trial type (3 levels: congruent, incongruent or passive) as within-subjects factor on the reported explicit sense of agency after each block.

### Results

#### Intentional binding check

A paired-samples *t*-test confirmed that intentional binding was larger on active compared to passive trials (407 versus 293ms, *t*(59) = -7.41, *p* < .001). This ensures that our implicit measurement of sense of agency was successful. Table 1 presents the participants’ average estimated delays as a function of block and actual delay (i.e., 500, 750, 1000 or 1250ms) for the correct active trials (excluding trials exceeding 2.5 *SD* of the overall mean RT) and the passive trials.

**Table 1:** Means (SD) for the estimated delays (in ms) in relation to block (EQ, MC, MI) and actual delay (500, 750, 1000 or 1250ms) for the active and passive trials.

		Active trials			Passive trials		
		Block			Block		
		EQ	MC	MI	EQ	MC	MI
Actual delay (ms)	500	365 (199.04)	381 (215.30)	362 (202.69)	451 (243.92)	442 (245.64)	462 (252.36)
	750	431 (226.34)	435 (228.86)	418 (219.39)	521 (271.00)	542 (281.28)	541 (287.26)
	1000	497 (253.58)	497 (267.92)	473 (245.68)	612 (319.79)	635 (298.32)	609 (326.32)
	1250	580 (296.26)	578 (296.13)	577 (296.99)	708 (369.62)	735 (368.97)	712 (378.26)

#### Trial-by-trial analyses

**Fig 2:**
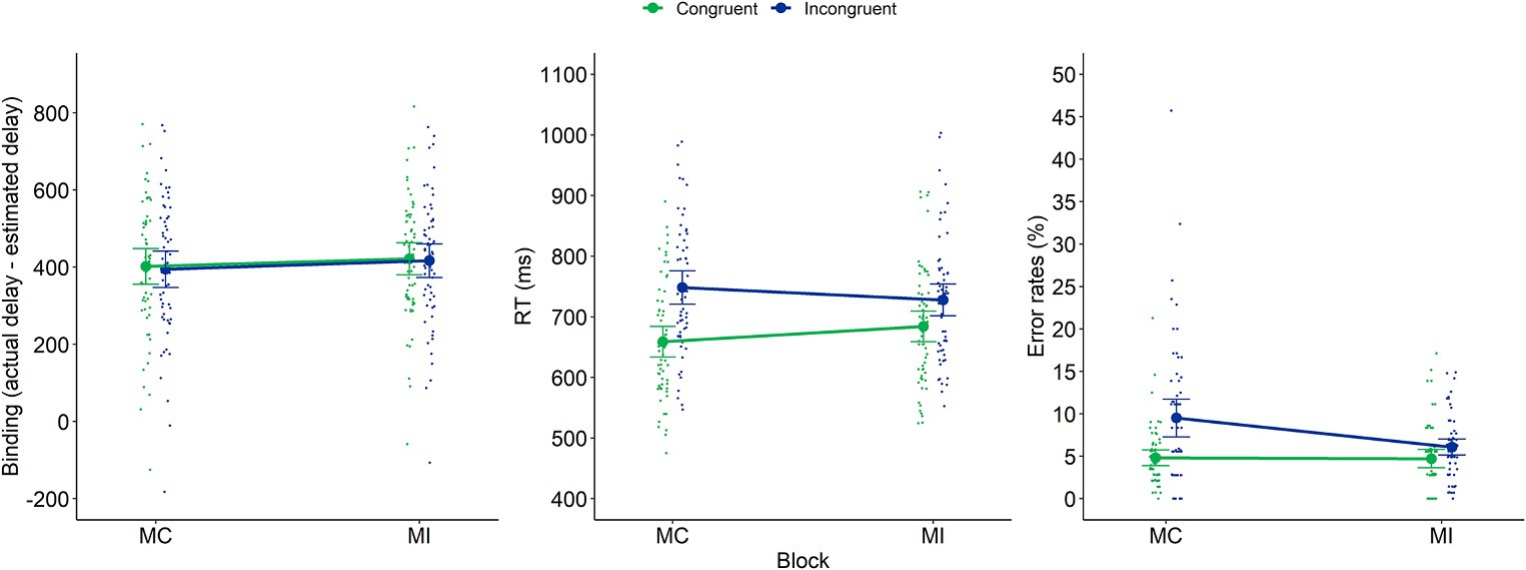
Intentional binding (left panel), RTs (middle panel) and error rates (right panel) of Experiment 1 as a function of previous congruency and current congruency. Dots represent mean RTs for each participant in each condition (thus, each participant is depicted four times on this graph, once for each Previous × Current Congruency condition). Error bars represent 95% confidence intervals.

These analyses were conducted on the EQ block. We conducted a 2x2 repeated measures analysis with Current and Previous trial congruency as within-subjects factors (both with 2 levels: congruent and incongruent) on intentional binding (in ms), RTs (in ms) and error rates (in %) as (separate) dependent variables. Means and *SD*s for each of these dependent variables in relation to current and previous congruency are reported in Table 2. The results are also depicted on Fig 2.

**Table 2:** Means (SD) for intentional binding (in ms), RTs (in ms) and error rates (in %) in relation to current and previous congruency (trial-by-trial analyses).

	Previous—Current trial congruency			
	CC	CI	IC	II
Binding	404 (180.10)	404 (169.50)	407 (178.75)	402 (177.13)
RT	659 (105.95)	729 (101.25)	670 (93.91)	710 (102.41)
Error rates	4.41 (4.20)	7.83 (6.18)	4.38 (3.91)	6.94 (6.10)

CC: congruent previous trial and congruent current trial; CI: congruent previous trial and incongruent current trial; IC: incongruent previous trial and congruent current trial; II: incongruent previous trial and incongruent current trial.

With regard to *intentional binding*, we found no significant main effects of current or previous congruency (resp. *F*(1,59) = 0.15, *p* = .70, *η*^2^_p_ = 0.003 and *F*(1,59) = 0.011, *p* = .92, *η*^2^_p_ < 0.001), nor a significant interaction (*F*(1,59) = 0.16, *p* = .69, *η*^2^_p_ = 0.003). This indicates that we observed no difference in intentional binding depending on current or previous congruency.

With regard to *RT*, we found a significant main effect of current congruency (*F*(1,59) = 90.65, *p* < .001, *η*^2^_p_ = 0.61) indicating that participants were slower on incongruent compared to congruent trials (on average 719.5ms versus 664.5ms). Additionally, we observed a significant interaction between current and previous congruency (*F*(1,59) = 8.85, *p* = .004, *η*^2^_p_ = 0.13). This interaction reflects a typical Gratton effect: a decreased congruency effect after an incongruent compared to a congruent trial (40ms versus 70ms). The observation of this Gratton effect ensures that our manipulation of current and previous congruency was successful in order to trigger trial-by-trial adaptations. The main effect of previous congruency did not reach significance (*F*(1,59) = 0.95, *p* = .33, *η*^2^_p_ = 0.016).

With regard to *error rates*, we only observed a significant main effect of current congruency (*F*(1,59) = 21.56, *p* < .001, *η*^2^_p_ = 0.27) indicating that participants made more errors on incongruent compared to congruent trials (on average 7.38% versus 4.39%). The main effect of previous congruency and the interaction between current and previous congruency did not reach significance (resp. *F*(1,59) = 1.32, *p* = .26, *η*^2^_p_ = 0.022 and *F*(1,59) = 0.77, *p* = .38, *η*^2^_p_ = 0.26).

As an exploratory analysis that was not included in our preregistration, we additionally conducted a 4x2x2 repeated measures analysis with Actual delay (4 levels: 500, 750, 1000 or 1250ms), Current and Previous trial congruency (both with 2 levels: congruent and incongruent) as within-subjects factors on intentional binding (in ms). We only observed a main effect of Actual delay (*F*(3,56) = 285.67, *p* < .001, *η*^2^_p_ = 0.94), with more intentional binding for longer delays (specifically, 132.5, 313, 501 and 670ms for the increasing delays). None of the other main effects or interactions reached significance.

#### Block analyses

**Fig 3:**
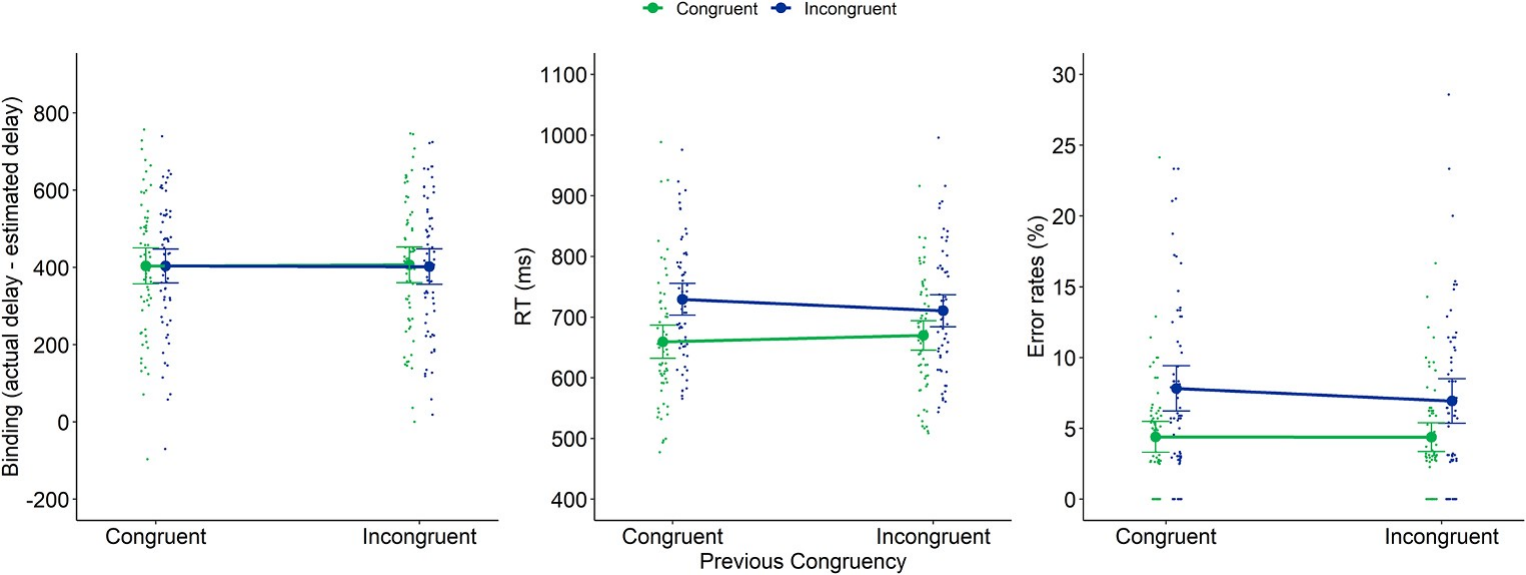
Intentional binding (left panel), RTs (middle panel) and error rates (right panel) of Experiment 1 as a function of Block and current congruency. Dots represent mean RTs for each participant in each condition (thus, each participant is depicted four times on this graph, once for each Block × Current Congruency condition). Error bars represent 95% confidence intervals.

These analyses were conducted on the MC and MI blocks. We conducted a 2x2 repeated measures analysis with Block (2 levels: MC and MI) and Current trial congruency (2 levels: congruent and incongruent) as within-subjects factors on intentional binding (in ms), RTs (in ms) and error rates (in %) as (separate) dependent variables. Means and *SD*s for each of these dependent variables in relation to current and previous congruency are reported in Table 3. The results are also depicted on Fig 3.

**Table 3:** Means (SD) for intentional binding (in ms), RTs (in ms) and error rates (in %) in relation to block and current congruency (block analyses).

	Block—Current trial congruency			
	MC_C	MC_I	MI_C	MI_I
Binding	402 (178.24)	395 (182.27)	422 (159.70)	417 (167.78)
RT	659 (97.38)	748 (106.66)	684 (97.61)	728 (101.94)
Error rates	4.83 (3.59)	9.51 (8.62)	4.71 (4.16)	6.09 (3.61)

MC_C: congruent trial in an MC block; MC_I: incongruent trial in an MC block; MI_C: congruent trial in an MI block; MI_I: incongruent trial in an MI block.

With regard to *intentional binding*, we found no significant main effect of congruency (*F*(1,59) = 1.56, *p* = .22, *η*^2^_p_ = 0.026), nor a significant interaction between block and congruency (*F*(1,59) = 0.048, *p* = .83, *η*^2^_p_ = 0.001). The main effect of block also did not reach significance, but showed a slight trend (*F*(1,59) = 3.00, *p* = .089, *η*^2^_p_ = 0.048). Indeed, we observed slightly more binding in the MI compared to the MC block, as hypothesized (419.5 versus 398.5ms).

With regard to *RT*, we found a significant main effect of congruency (*F*(1,59) = 286.29, *p* < .001, *η*^2^_p_ = 0.83) indicating that participants were slower on incongruent compared to congruent trials (on average 738ms versus 671.5ms). Additionally, we observed a significant interaction between block and congruency (*F*(1,59) = 45.80, *p* < .001, *η*^2^_p_ = 0.44). This interaction reflects a typical proportion congruency effect: a decreased congruency effect in an MI block compared to an MC block (44ms versus 89ms). The observation of this proportion congruency effect ensures that our manipulation of block was successful in order to trigger blockwise adaptations. The main effect of block did not reach significance (*F*(1,59) = 0.056, *p* = .81, *η*^2^_p_ = 0.001).

With regard to *error rates*, we observed a significant main effect of congruency (*F*(1,59) = 31.93, *p* < .001, *η*^2^_p_ = 0.35) indicating that participants made more errors on incongruent compared to congruent trials (on average 7.80% versus 4.77%). A main effect of block was also observed (*F*(1,59) = 10.91, *p* = .002, *η*^2^_p_ = 0.16), indicating that participants made more errors on the MC block compared to the MI block (7.17% versus 5.40%). Finally, the interaction between block and congruency also reached significance (resp. *F*(1,59) = 1.32, *p* = .26, *η*^2^_p_ = 0.022), again indicating a proportion congruency effect: a decreased congruency effect in an MI block compared to an MC block (1.38% versus 4.68%).

As an exploratory analysis that was not included in our preregistration, we additionally conducted a 4x2x2 repeated measures analysis with Actual delay (4 levels: 500, 750, 1000 or 1250ms), Block (2 levels: MC and MI) and Congruency (2 levels: congruent and incongruent) as within-subjects factors on intentional binding (in ms). We only observed a main effect of Actual delay (*F*(3,56) = 372.89, *p* < .001, *η*^2^_p_ = 0.95), with more intentional binding for longer delays (specifically, 128, 328, 518.5 and 682ms for the increasing delays). None of the other main effects or interactions reached significance.

#### Post-block subjective effort and agency analyses

With regard to *subjective effort* experienced during each block (EQ, MC or MI), we conducted a repeated measures analysis with block (3 levels: EQ, MC or MI) as within-subjects factor on the reported experienced effort after each block. We observed no significant differences between the three blocks (*F*(2,58) = 0.14, *p* = .87, *η*^2^_p_ = 0.005). Indeed, the reported experienced effort was very similar in the EQ, MC and MI blocks (resp. 6.44, 6.53 and 6.36).

With regard to the *subjective sense of agency* experienced for congruent, incongruent and passive trials, we conducted a repeated measures analysis with trial type (3 levels: congruent, incongruent or passive) as within-subjects factor on the reported explicit sense of agency after each block. We observed significant differences between the experienced agency over congruent, incongruent and passive trials (*F*(2,58) = 16.83, *p* < .001, *η*^2^_p_ = 0.37). Specifically, participants reported a higher sense of agency over incongruent trials (4.81) compared to congruent (4.2, *F*(1,59) = 24.61, *p* < .001, *η*^2^_p_ = 0.29) or passive trials (4.1, *F*(1,59) = 7.98, *p* = .006, *η*^2^_p_ = 0.12). The explicit sense of agency did not differ between congruent and passive trials (4.1, *F*(1,59) = 0.12, *p* = .73, *η*^2^_p_ = 0.02). Note that this effect did not interact with block: a repeated measures analysis with trial type (3 levels: congruent, incongruent or passive) and block (3 levels: EQ, MC or MI) as within-subjects factors again only revealed a main effect of trial type (*F*(2,58) = 16.83, *p* < .001, *η*^2^_p_ = 0.37).

### Discussion

Experiment 1 confirmed that our manipulation of cognitive control was successful: incongruent trials led to a slower and more erroneous response than congruent trials (i.e., flanker effect). After an incongruent trial, participants also increased their exertion of cognitive control compared to after a congruent trial (i.e., Gratton effect). Finally, when conflict could be anticipated (i.e., MI block), the flanker effect was reduced (i.e., proportion congruency effect). Our implicit intentional binding measure also seemed to be successful: participants reported shorter time intervals on active trials compared to passive trials. However, we were unable to confirm the results of previous studies: we observed no decrease in binding on incongruent trials, nor an increase in binding after incongruent trials. We did observe a slight trend towards increased binding in a context where conflict could be anticipated (i.e., MI block).

Interestingly, our explicit measure of sense of agency, administered after each block, did indicate that participants experienced more agency over incongruent trials than over congruent trials. According to the two-step account of agency, the implicit and explicit approaches to measure sense of agency, capture separate aspects of the sense of agency, namely the implicit feeling of agency and the explicit judgement of agency [27,28]. The implicit feeling of agency stems from a low-level comparator involving motor action planning and prediction, and sensory feedback processes. The explicit judgement of agency relies on higher-order causality judgements based on contextual factors and beliefs [29]. Furthermore, although correlations between intentional binding and subjective sense of agency have been reported [26], whether binding truly reflects sense of agency is debated [30,31]. Based on this, we decided to conduct the experiment again, but now using an explicit measure of sense of agency on each trial (see also [17]).

## Experiment 2

### Method

#### Participants

Sixty participants were recruited from the Experiment Management System of the Katholieke Universiteit Leuven. They received course credit or a monetary reward for participation. All participants provided written informed consent. None of the them had participated in Experiment 1. All of them had normal or corrected-to-normal eyesight, were not colorblind and were able to operate a keyboard and mouse. We used the following exclusion criteria for participants: response times and/or error rates above 20%. Two participants did not meet these criteria based on their response time. Thus, 58 participants were included for the analyses (9 males, mean age = 20.67, *SD* = 4.57, range 17–40). This study was approved by the Social and Societal Ethics Committee (SMEC) from KU Leuven (G-2019 05 1652). The study was also preregistered on OSF (osf.io; doi:10.17605/OSF.IO/EM3GQ) and the raw data can be retrieved from https://osf.io/em3gq/ (doi: 10.17605/OSF.IO/EM3GQ).

#### Apparatus, design, procedure and statistical analyses

**Fig 4:**
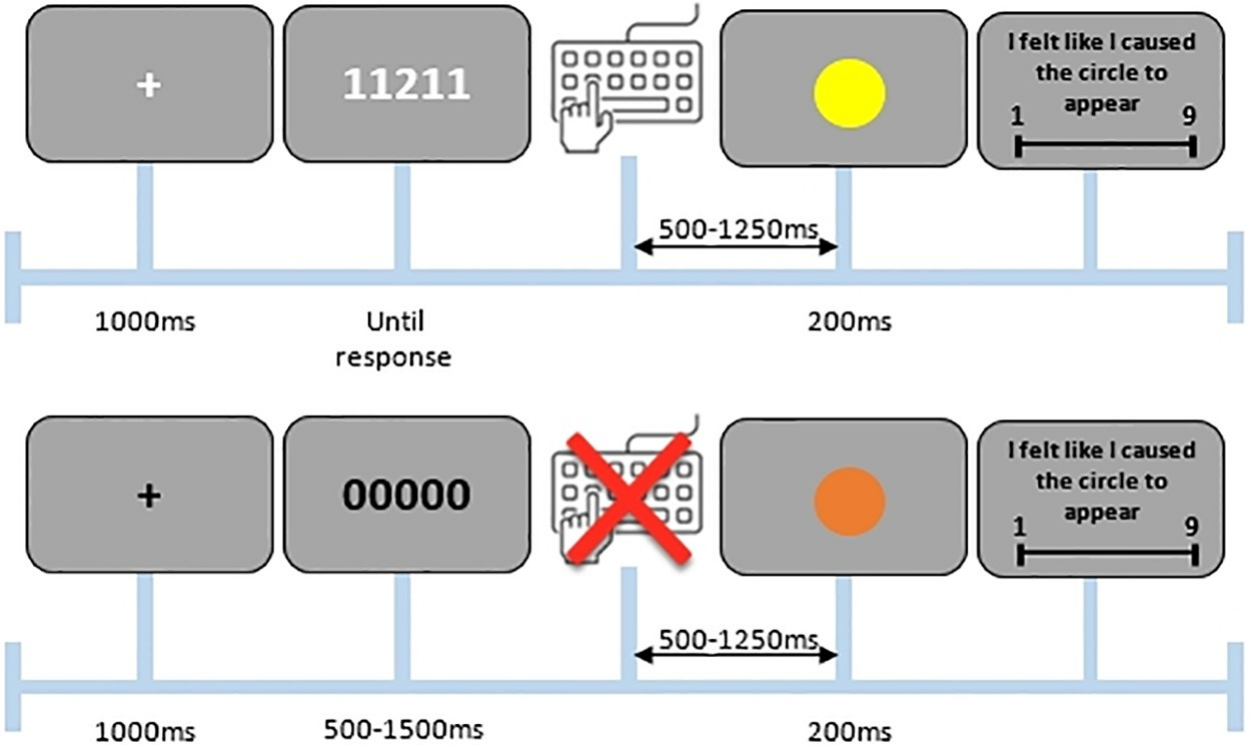
Example of an active (top) and passive (bottom) trial in Experiment 2.

The apparatus, design and procedure were identical to Experiment 1, except for the following changes. Instead of using an implicit measure of sense of agency, as we did in Experiment 1 (i.e., intentional binding based on the Interval Reproduction Task), we now used an explicit measure of sense of agency. Specifically, after the disappearance of the colored circle, participants now had to indicate their explicit sense of agency (i.e., “I felt like I caused the circle to appear”) using a 9-point scale ranging from “strongly disagree” to “strongly agree” [17]. This was the case for both active and passive trials. For a schematic overview of both trial types, see Fig 4. Statistical analyses were also the same as in Experiment 1, except that this explicit sense of agency was now used as dependent variable instead of intentional binding.

Note that, as explained in Experiment 1, we did not pool all trials across blocks to assess the general effect of current trial congruency on sense of agency ratings. However, an additional exploratory repeated measures analysis across all blocks with current trial congruency as within-subjects factor revealed that the main effect of congruency on sense of agency ratings was not significant (*F*(1,57) = 1.81, *p* = .18, *η*^2^_p_ = 0.031).

Active trials exceeding 2.5 *SD* of the overall mean RT were excluded from all analyses (i.e., 1.9%). For RT and intentional binding analyses, erroneous active flanker trials were removed (i.e., 4.5%). For trial-by-trial analyses assessing hypotheses 1 and 2, the first trial of the block (0.46%) and trials following an erroneous flanker trial (i.e., 3.8%) were also excluded in the EQ block. For one participant, the sense of agency and effort ratings after each block were not recorded. Therefore, this participant was excluded from the post-block analyses.

### Results

#### Sense of agency rating check

A paired-samples *t*-test confirmed that the reported sense of agency was larger on active compared to passive trials (5.51 versus 2.56ms, *t*(57) = -9.79, *p* < .001). This ensures that our explicit measurement of sense of agency was successful. Table 4 presents the participants’ sense of agency ratings as a function of block and delay (i.e., 500, 750, 1000 or 1250ms) for the correct active trials (excluding trials exceeding 2.5 *SD* of the overall mean RT) and the passive trials.

**Table 4:** Means (SD) for the sense of agency ratings (score between 1–9) in relation to block (EQ, MC, MI) and delay (500, 750, 1000 or 1250ms) for the active and passive trials.

		Active trials			Passive trials		
		Block			Block		
		EQ	MC	MI	EQ	MC	MI
Actual delay (ms)	500	5.68 (2.90)	5.70 (2.92)	5.85 (2.82)	2.53 (2.66)	2.62 (2.68)	2.53 (2.63)
	750	5.43 (2.91)	5.64 (2.86)	5.53 (2.79)	2.46 (2.60)	2.58 (2.63)	2.68 (2.78)
	1000	5.43 (2.92)	5.47 (2.90)	5.45 (2.82)	2.57 (2.68)	2.41 (2.42)	2.50 (2.66)
	1250	5.16 (2.92)	5.32 (2.93)	5.36 (2.79)	2.71 (2.84)	2.58 (2.67)	2.42 (2.68)

#### Trial-by-trial analyses

**Fig 5:**
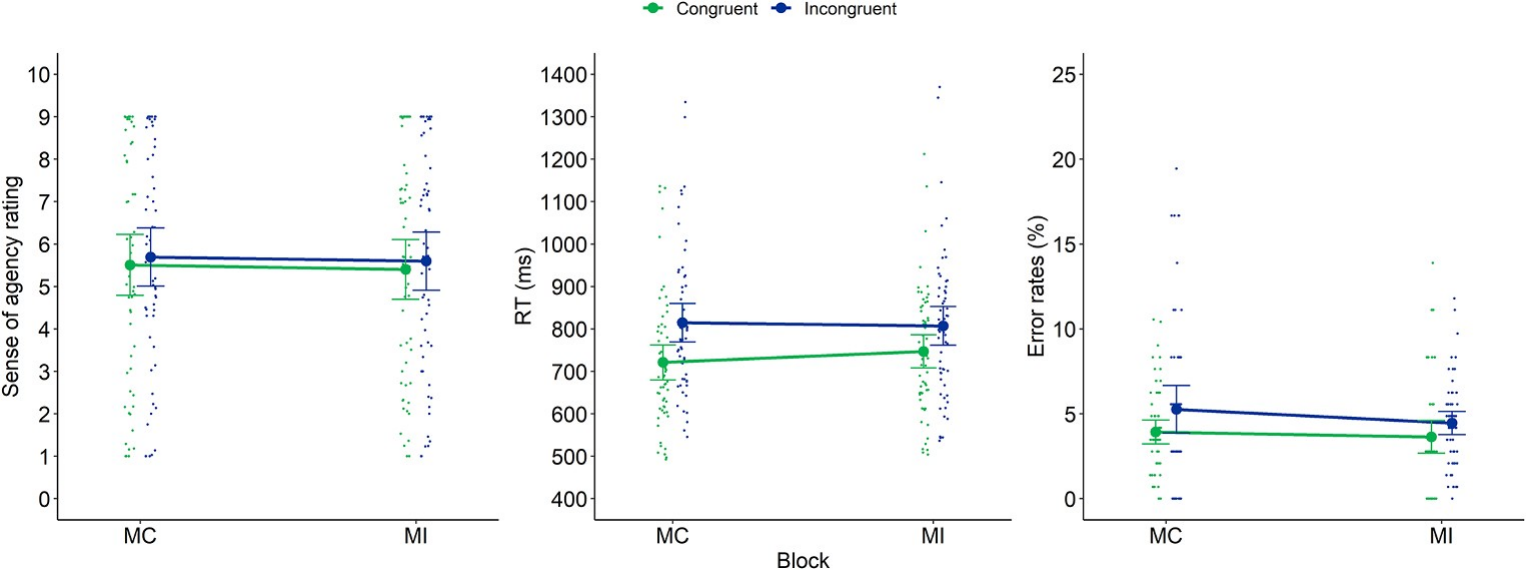
Sense of agency ratings (left panel), RTs (middle panel) and error rates (right panel) of Experiment 2 as a function of previous congruency and current congruency. Dots represent mean RTs for each participant in each condition (thus, each participant is depicted four times on this graph, once for each Previous × Current Congruency condition). Error bars represent 95% confidence intervals.

These analyses were conducted on the EQ block. We conducted a 2x2 repeated measures analysis with Current and Previous trial congruency as within-subjects factors (both with 2 levels: congruent and incongruent) on sense of agency (score on a scale from 1 to 9), RTs (in ms) and error rates (in %) as (separate) dependent variables. Means and *SD*s for each of these dependent variables in relation to current and previous congruency are reported in Table 5. The results are also depicted on Fig 5.

**Table 5:** Means (SD) for sense of agency (rating from 1 to 9), RTs (in ms) and error rates (in %) in relation to current and previous congruency (trial-by-trial analyses).

	Previous—Current trial congruency			
Means (*SD*)	CC	CI	IC	II
Sense of Agency	5.38 (2.73)	5.51 (2.66)	5.40 (2.71)	5.51 (2.65)
RT	727 (165.33)	810 (182.57)	730 (140.70)	790 (173.53)
Error rates	4.67 (4.74)	4.68 (4.66)	4.22 (3.76)	4.79 (3.81)

CC: congruent previous trial and congruent current trial; CI: congruent previous trial and incongruent current trial; IC: incongruent previous trial and congruent current trial; II: incongruent previous trial and incongruent current trial.

With regard to *sense of agency*, we found no significant main effect of previous congruency (*F*(1,57) = 0.045, *p* = .83, *η*^2^_p_ = 0.001), nor a significant interaction (*F*(1,57) = 0.16, *p* = .69, *η*^2^_p_ = 0.003). The main effect of current congruency was not significant, but showed a trend (*F*(1,57) = 3.29, *p* = .075, *η*^2^_p_ = 0.055): incongruent trials received a slightly higher agency rating than congruent trials (5.51 versus 5.39).

With regard to *RT*, we found a significant main effect of current congruency (*F*(1,57) = 41.57, *p* < .001, *η*^2^_p_ = 0.42) indicating that participants were slower on incongruent compared to congruent trials (on average 800ms versus 728.5ms). The main effect of previous congruency did not reach significance (*F*(1,57) = 0.75, *p* = .39, *η*^2^_p_ = 0.013). We also did not observe a significant interaction between current and previous congruency (*F*(1,57) = 1.82, *p* = .18, *η*^2^_p_ = 0.031). This implies that we did not observe a typical Gratton effect: the congruency effect was not significantly decreased after an incongruent compared to a congruent trial (60ms versus 83ms).

With regard to *error rates*, we observed no significant effects (all *p* > .55).

As an exploratory analysis that was not included in our preregistration, we additionally conducted a 4x2x2 repeated measures analysis with Delay (4 levels: 500, 750, 1000 or 1250ms), Current and Previous trial congruency (both with 2 levels: congruent and incongruent) as within-subjects factors on sense of agency ratings (score between 1–9). We observed a main effect of Actual delay (*F*(3,55) = 3.41, *p* = .024, *η*^2^_p_ = 0.16), with slightly decreasing sense of agency for longer delays (specifically, 5.63, 5.51, 5.38 and 5.28 for the increasing delays). We also observed a main effect of Current trial congruency (*F*(1,57) = 4.28, *p* = .043, *η*^2^_p_ = 0.070) with a slightly higher sense of agency for incongruent trials compared to congruent trials (5.51 and 5.38, respectively). None of the other main effects or interactions reached significance.

#### Block analyses

**Fig 6:**
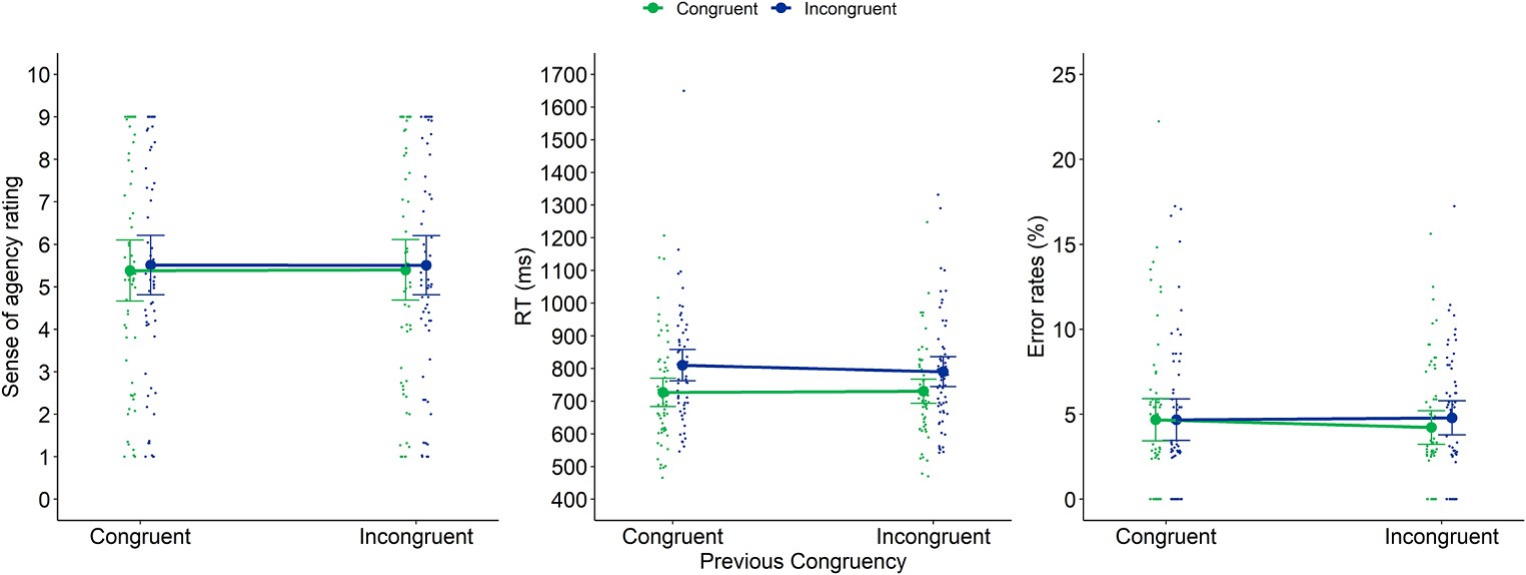
Sense of agency ratings (left panel), RTs (middle panel) and error rates (right panel) of Experiment 2 as a function of Block and current congruency. Dots represent mean RTs for each participant in each condition (thus, each participant is depicted four times on this graph, once for each Block × Current Congruency condition). Error bars represent 95% confidence intervals.

These analyses were conducted on the MC and MI blocks. We conducted a 2x2 repeated measures analysis with Block (2 levels: MC and MI) and Current trial congruency (2 levels: congruent and incongruent) as within-subjects factors sense of agency (score on a scale from 1 to 9), RTs (in ms) and error rates (in %) as (separate) dependent variables. Means and *SD*s for each of these dependent variables in relation to current and previous congruency are reported in Table 6. The results are also depicted on Fig 6.

**Table 6:** Means (SD) for sense of agency (rating from 1 to 9), RTs (in ms) and error rates (in %) in relation to block and current congruency (block analyses).

	Block—Current trial congruency			
Means (*SD*)	MC_C	MC_I	MI_C	MI_II
Sense of agency	5.51 (2.73)	5.69 (2.60)	5.40 (2.68)	5.60 (2.60)
RT	721 (157.33)	815 (171.56)	747 (148.94)	807 (173.99)
Error rates	3.93 (2.65)	5.27 (5.32)	3.64 (3.65)	4.45 (2.60)

MC_C: congruent trial in an MC block; MC_I: incongruent trial in an MC block; MI_C: congruent trial in an MI block; MI_I: incongruent trial in an MI block.

With regard to *sense of agency*, we found a significant main effect of congruency (*F*(1,57) = 6.94, *p* = .011, *η*^2^_p_ = 0.11), indicating that participants reported slightly more agency on incongruent compared to congruent trials (5.64 versus 5.46). The main effect of block did not reach significance (*F*(1,57) = 0.42, *p* = .52, *η*^2^_p_ = 0.007), nor did the interaction between block and congruency (*F*(1,57) = 0.024, *p* = .88, *η*^2^_p_ < 0.001).

With regard to *RT*, we found a significant main effect of congruency (*F*(1,57) = 113.85, *p* < .001, *η*^2^_p_ = 0.67) indicating that participants were slower on incongruent compared to congruent trials (on average 811ms versus 734ms). Additionally, we observed a significant interaction between block and congruency (*F*(1,57) = 5.52, *p* = .022, *η*^2^_p_ = 0.088). This interaction reflects a typical proportion congruency effect: a decreased congruency effect in an MI block compared to an MC block (60ms versus 94ms). The observation of this proportion congruency effect ensures that our manipulation of block was successful in order to trigger blockwise adaptations. The main effect of block did not reach significance (*F*(1,57) = 0.56, *p* = .46, *η*^2^_p_ = 0.010).

With regard to *error rates*, we observed a significant main effect of congruency (*F*(1,57) = 6.65, *p* = .013, *η*^2^_p_ = 0.10) indicating that participants made more errors on incongruent compared to congruent trials (on average 4.86% versus 3.78%). The main effect of block (*F*(1,57) = 2.19, *p* = .14, *η*^2^_p_ = 0.037) and the interaction between block and congruency (*F*(1,57) = 0.50, *p* = .48, *η*^2^_p_ = 0.009) did not reach significance.

As an exploratory analysis that was not included In our preregistration, we additionally conducted a 4x2x2 repeated measures analysis with Actual delay (4 levels: 500, 750, 1000 or 1250ms), Block (2 levels: MC and MI) and Congruency (2 levels: congruent and incongruent) as within-subjects factors on sense of agency ratings (score between 1–9). We observed a main effect of Actual delay (*F*(3,55) = 6.32, *p* = .001, *η*^2^_p_ = 0.269), with slightly decreasing agency ratings for longer delays (specifically, 5.77, 5.61, 5.48 and 5.35 for the increasing delays). We also observed a main effect of Congruency (*F*(1,57) = 6.61, *p* = .013, *η*^2^_p_ = 0.10), with a slightly higher sense of agency for incongruent trials compared to congruent trials (5.65 and 5.46, respectively). None of the other main effects or interactions reached significance.

#### Post-block subjective effort and agency analyses

With regard to *subjective effort* experienced during each block (EQ, MC or MI), we conducted a repeated measures analysis with block (3 levels: EQ, MC or MI) as within-subjects factor on the reported experienced effort after each block. We observed no significant differences between the three blocks (*F*(2,55) = 0.82, *p* = .44, *η*^2^_p_ = 0.029). Indeed, the reported experienced effort was very similar in the EQ, MC and MI blocks (resp. 4.86, 4.87 and 5.20).

With regard to the *subjective sense of agency* experienced for congruent, incongruent and passive trials, we conducted a repeated measures analysis with trial type (3 levels: congruent, incongruent or passive) as within-subjects factor on the reported explicit sense of agency after each block. We observed significant differences between the experienced agency over congruent, incongruent and passive trials (*F*(2,55) = 48.90, *p* < .001, *η*^2^_p_ = 0.64). Specifically, participants reported a higher sense of agency over incongruent trials (5.71) compared to congruent (5.03, *F*(1,56) = 18.78, *p* < .001, *η*^2^_p_ = 0.25) or passive trials (2.63, *F*(1,56) = 96.65, *p* < .001, *η*^2^_p_ = 0.63). The explicit sense of agency also differed between congruent and passive trials (*F*(1,56) = 60.03, *p* < .001, *η*^2^_p_ = 0.52). Note that this effect did not interact with block: a repeated measures analysis with trial type (3 levels: congruent, incongruent or passive) and block (3 levels: EQ, MC or MI) as within-subjects factors again only revealed a main effect of trial type (*F*(2,55) = 45.32, *p* < .001, *η*^2^_p_ = 0.62).

### Discussion

Next to confirming again that our manipulation of cognitive control and our measurement of sense of agency were successful, we only obtained a small effect of congruency on the sense of agency, both in the trial-by-trials and the block analyses: incongruent trials received a slightly higher agency rating than congruent trials. This is in line with the post-block reported explicit sense of agency in Experiment 1, but contrary to our hypothesis. In the general discussion we will elaborate this further.

## General discussion

The aim of this study was to examine the effect of cognitive effort on sense of agency. We were interested in shedding light on the contradictory literature about the facilitative or/and detrimental nature of cognitive effort on sense of agency. Based on previous studies we suggested that temporal aspects might determine when effort facilitates or impedes sense of agency. We formulated concrete hypotheses. First, during a block of trials where effort is required unpredictably, encountering a high-effort trial would lead to a decreased sense of agency *on that same trial*, but an increased sense of agency *on the next trial* compared to a low-effort trial. Second, in a block of trials where effort can be anticipated (i.e., frequent high-effort trials), sense of agency would be increased compared to a block of trials where effort cannot be anticipated (i.e., scarce high-effort trials). Furthermore, we aimed to explore the effect of cognitive effort on both implicit and explicit measures of sense of agency. We used a flanker task while also varying the proportion of conflict present in a block of trials, creating three conditions (MC, MI and EQ block) in which the required cognitive control (and hence effort) varied.

Our results showed that our manipulations of cognitive control, intentional binding (Experiment 1) and sense of agency (Experiment 2) were successful. With regards to the trial-by-trial analyses, and contrarily to our expectations, we observed no decreased intentional binding or sense of agency *on* incongruent trials, nor an increased intentional binding or sense of agency *after* incongruent trials. Remarkably, in the trial-by-trial analyses of Experiment 2, we observed a trend towards an increase of sense of agency on current incongruent trials compared to congruent trials (which became significant when actual delay was taken into account). This increased sense of agency for incongruent trials was also observed in the block analyses of Experiment 2 and in the post-block analyses of both experiments. With regards to the block analyses of Experiment 1, we observed a slight trend towards increased intentional binding in a context where conflict could be anticipated (i.e., MI block) compared to a context where conflict was scarce (i.e., MC block). This trend was not present for sense of agency in Experiment 2.

Our results are not in line with previous studies observing lower sense of agency for incongruent compared to congruent trials (e.g. [17,19]). A striking difference between our study and these previous studies, is the interval between action and outcome and hence when participants are asked to introspect about their sense of agency. Previous studies used short intervals (ranging from 100 to 500ms), whereas our intervals ranged from 500 to 1250ms. We chose these longer intervals (see also [23]) to be able to use the spacebar Interval Reproduction Task, as a measure of intentional binding, in Experiment 1. But the moment when sense of agency is tapped into, might be crucial. After shorter action-outcome intervals, perhaps the experience of conflict is still predominant, leading to smaller agency ratings on incongruent trials. After longer intervals, perhaps more metacognitive processes might come into play, leading participants to reappraise their action as an accomplishment of successfully solving an incongruent trials, leading to higher agency ratings on incongruent trials. It has already been shown that conflict experience precedes metacognitive experience, which might only occur at a later time [32]. In order to further elucidate this, we should include a broader range of action-outcome intervals in the experimental design.

Interestingly, our results are in line with Damen et al. [33], who, using quite a different free choice priming task, found that clearly visible incongruent primes increased agency compared to congruent primes. Based on this observation, another possible explanation for the discrepancy in results might be the exact way in which sense of agency is probed. Both in our study and the study of Damen et al. [33] we particularly asked participants whether they felt they had *caused* the outcome. In contrast, Sidarus and Haggard [17] and Wang et al. [19] asked participants how much *control* they felt over the outcome that was triggered by their actions. This subtle difference in instructions, might have shifted participants’ focus from how much control they felt (which arguably might be larger for congruent trials) to how much causality they experienced (which might be higher for incongruent trials which require the overruling of habitual behavior). A similar argument can be made for the post-block assessment of sense of agency. In previous studies, participants had to rank the colored circles and rate their sense of control for each of them after each block of trials. In our study, in contrast, participants reflected on the extent to which they felt they had caused the colored circles to appear on congruent, incongruent and passive trial specifically. Next to the difference between the focus on control versus causality, additionally, we specifically highlighted the aspect of congruency here. This in turn might have led to a reappraisal of their actions in response to the clearly more difficult, incongruent trials. A follow-up study manipulating instructions might be very informative with regards to the role of even subtle nuances in sense of agency instructions.

In this study, we departed from the view that sense of agency and effort are tightly related and that a subjective experience of effort might be a prerequisite for any feeling of agency or causality. From this, we speculated that experimental manipulations of cognitive effort should also influence the accompanying sense of agency. However, whether our manipulation of effort was successful, is doubtful. Although only assessed after each block and only per block type and not per trial-type, participants reported equal experiences of effort for MC, MI and EQ blocks in both experiments. Thus, although our manipulation of cognitive control was successful (e.g., reduced congruency effects in MI compared to MC blocks, indicative of increased cognitive control in MI blocks), this might not have been accompanied by differences in experienced effort. Indeed, the constructs of cognitive effort and cognitive control are not identical, and the possibility of effortless exertion of cognitive control (i.e., “flow”) has been suggested [1]. Alternatively, even though it is expected that in an MI block the overall cognitive control, and hence effort experience, is larger, perhaps the intense effort experienced on the rare incongruent trials in an MC block cancelled out the effort differences at the block level. Finally, it could also be the case that the experienced differences in effort in our experiments were too subtle to be crudely captured only after a block of trials. Indeed, despite the absence of crude differences in effort between blocks, we still observed a trend of a difference in intentional binding between blocks. On the other hand, the lack of clear effort differences between the blocks, might also explain why our block-based results are minimal. Contrary to what we expected, we did not find an increased explicit sense of agency in a context where conflict could be anticipated (i.e., MI block) compared to a context where conflict was rare (i.e., MC block), but we did observe a slight trend towards increased intentional binding in the MI block compared to the MC block. These results could be linked to the fact that the MI block probably was not effortful enough. In any case, replicating the experiments using a task that induces stronger differences in experienced effort between the different conditions (e.g., Stroop task), and regularly assessing the experienced effort for different trial types and blocks seems advisable.

Our findings seem to imply a dissociation between our intentional binding and sense of agency measures. First, despite no apparent crude difference in experienced effort, the explicit sense of agency measure was still able to pick up some (trends towards) differences between experienced sense of agency for congruent and incongruent trials, whereas the intentional binding measure was not. It could be that intentional binding is a less sensitive measure (especially when there are no strong differences in experienced effort). Alternatively, the lack of clear effort differences between congruent and incongruent trials, might also inhibit differences at the assumed implicit, pre-reflective level of sense of agency, but not the at the explicit reflective, inferential, belief-like level [28]. Second, across both experiments, the delay between the action and the outcome had an effect on our measures of sense of agency: participants showed more intentional binding, but lower sense of agency ratings when the delay increased (i.e., 500, 750, 1000 or 1250ms). This is in line with previous studies. Temporal binding measured using interval estimation is typically larger for longer intervals ([18,25,34,35] but see [26] for the reversed result), whereas higher agency ratings are often found for shorter intervals [26,36–39]. We are not the first to highlight differences between different measures of sense of agency (e.g., [25,26]). Recently, Imaizumi and Tanno [26] have suggested that intentional binding measured using time interval might originate from a different mechanism. Suzuki et al. [31] even showed that intentional binding might not necessarily reflect sense of agency, but might be accounted for by multisensory causal binding, without necessarily being related to intention or agency.

We sought to combine methods typically used in intentional binding tasks, with methods employed to study the effect of effort on explicit sense of agency. More specifically, in line with most binding studies (see [40]), we compared an active condition to a passive condition to assess the baseline level of intentional binding. This differs from previous studies that have assessed the effect of action selection fluency (effort) on explicit sense of agency, which typically do not include passive trials (e.g., [17]). While comparing active and passive trials remains the most common way of assessing intentional binding, it should be noted that this has been criticised for not adequately controlling for other processes that differ between these two types of trials (see [40]). In addition, the inclusion of passive trials in our studies, leading to a clearly felt difference in agency between active and passive trials, might have obscured more subtle differences between the different active trial types (e.g., congruent and incongruent trials). Therefore, an experiment presenting only active trials might be useful to expose perhaps elusive differences in agency between active trial types.

The current study examined the effect of cognitive effort on sense of agency using explicit and implicit measures of sense of agency. We showed that trials requiring the exertion of more cognitive control lead to a higher sense of agency. This result was contrasted to previous studies to establish potential reasons for this contradictory finding. Future studies should ensure that conditions are sufficiently effortful, use a broad range of action-outcome intervals and contrast (even subtle) different ways of probing sense of agency.
