# Correction: The effect of cognitive effort on the sense of agency

**DOI:** 10.1371/journal.pone.0269055

**Published:** 2022-05-23

**Authors:** Eva Van den Bussche, Maryna Alves, Yannick P. J. Murray, Gethin Hughes

The images for Figs 2 and 3 are incorrectly switched. The image that appears as Fig 2 should be Fig 3, and the image that appears as Fig 3 should be Fig 2. The figure captions appear in the correct order.

Additionally, the images for Figs 5 and 6 are incorrectly switched. The image that appears as Fig 5 should be Fig 6, and the image that appears as Fig 6 should be Fig 5. The figure captions appear in the correct order.

Please see the figures with the correct captions below.

**Fig 2:**
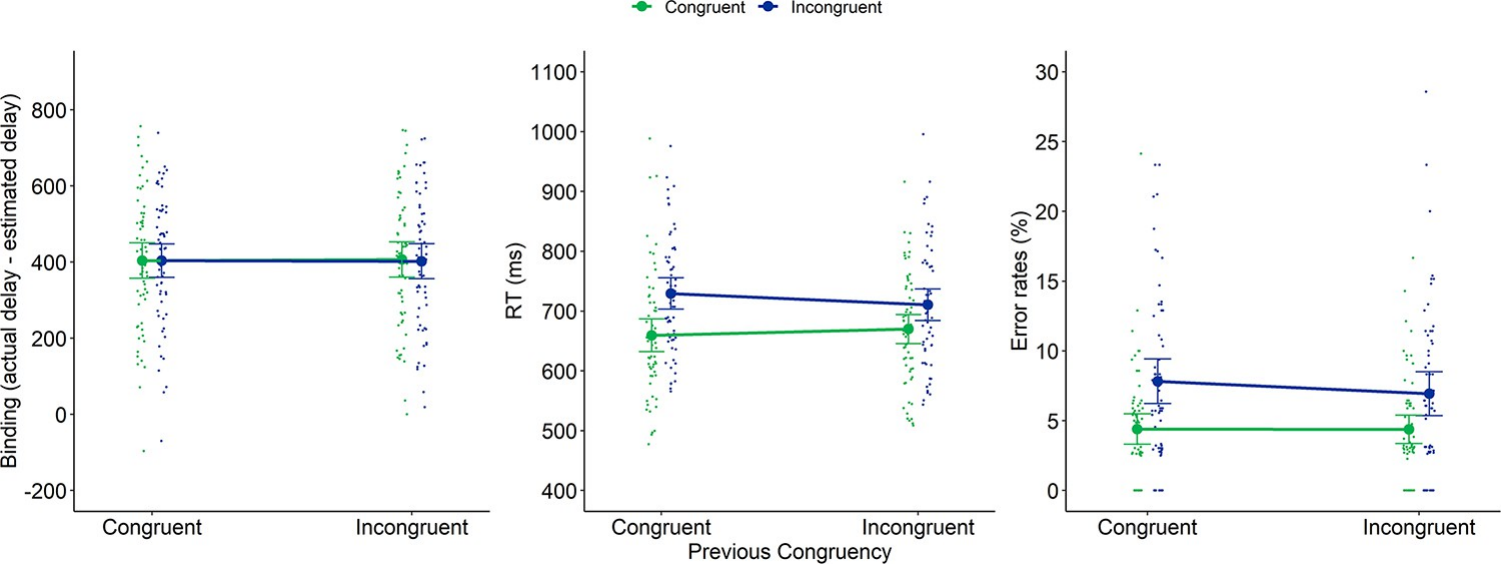
Intentional binding (left panel), RTs (middle panel) and error rates (right panel) of Experiment 1 as a function of previous congruency and current congruency. Dots represent mean RTs for each participant in each condition (thus, each participant is depicted four times on this graph, once for each Previous × Current Congruency condition). Error bars represent 95% confidence intervals.

**Fig 3:**
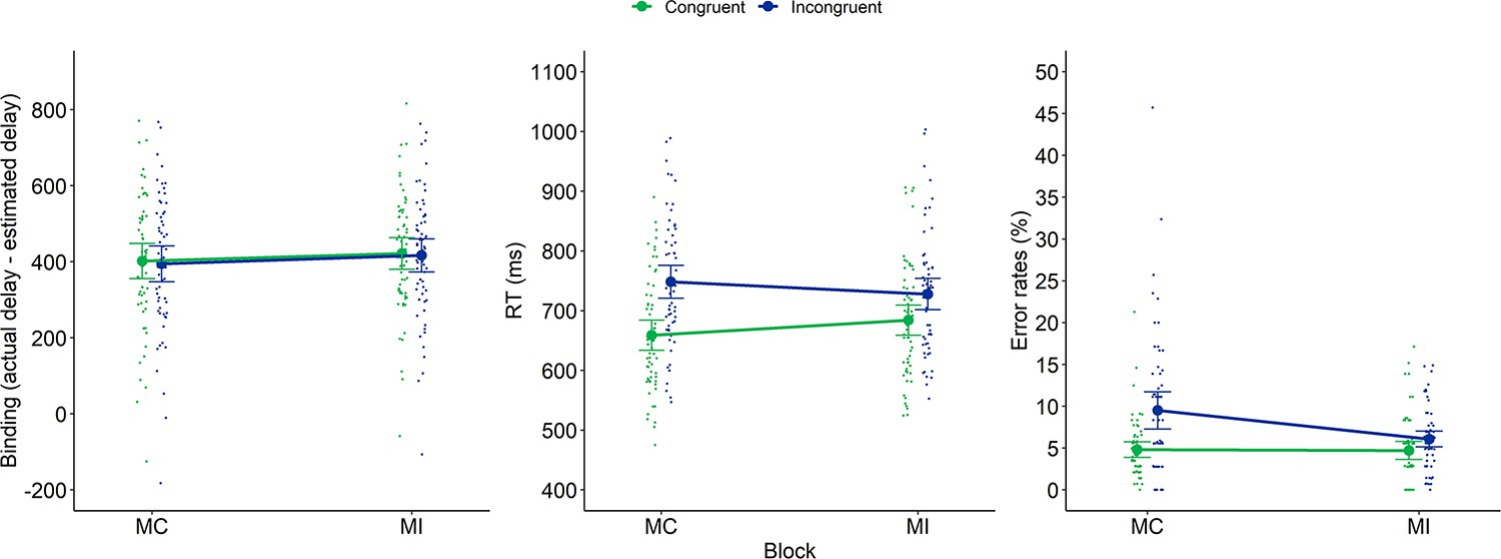
Intentional binding (left panel), RTs (middle panel) and error rates (right panel) of Experiment 1 as a function of Block and current congruency. Dots represent mean RTs for each participant in each condition (thus, each participant is depicted four times on this graph, once for each Block × Current Congruency condition). Error bars represent 95% confidence intervals.

**Fig 5:**
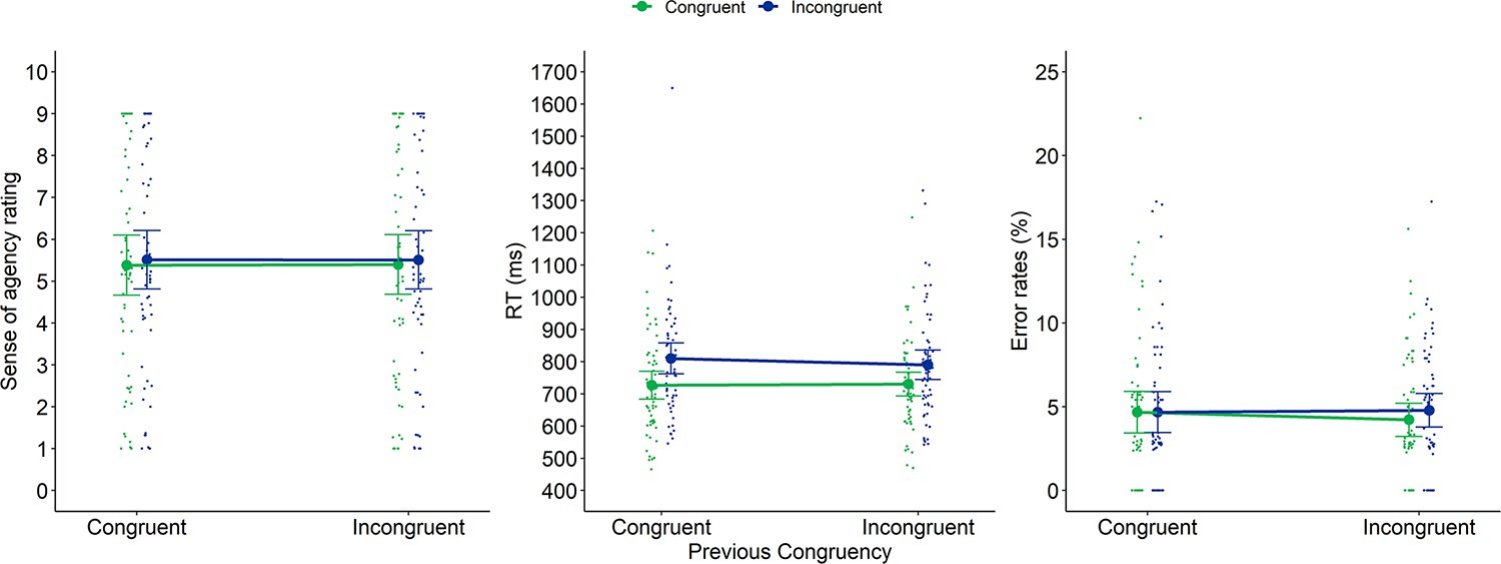
Sense of agency ratings (left panel), RTs (middle panel) and error rates (right panel) of Experiment 2 as a function of previous congruency and current congruency. Dots represent mean RTs for each participant in each condition (thus, each participant is depicted four times on this graph, once for each Previous × Current Congruency condition). Error bars represent 95% confidence intervals.

**Fig 6:**
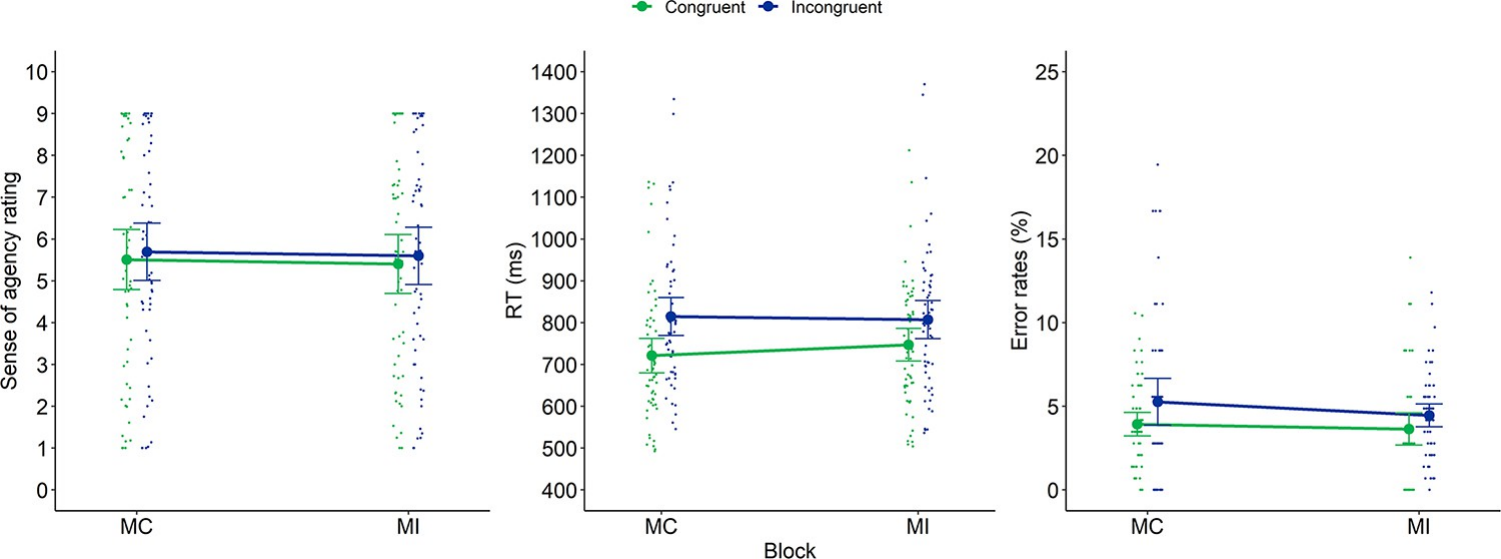
Sense of agency ratings (left panel), RTs (middle panel) and error rates (right panel) of Experiment 2 as a function of Block and current congruency. Dots represent mean RTs for each participant in each condition (thus, each participant is depicted four times on this graph, once for each Block × Current Congruency condition). Error bars represent 95% confidence intervals.
